# The Effect of Liming and Sewage Sludge Application on Heavy Metal Speciation in Soil

**DOI:** 10.1007/s00128-016-1984-3

**Published:** 2016-11-24

**Authors:** Elżbieta Malinowska

**Affiliations:** Department of Grassland and Landscape Architecture, Siedlce University of Natural Sciences and Humanities, B. Prusa 14 Street, 08–110 Siedlce, Poland

**Keywords:** Lead, Chromium, Nickel, Sewage sludge, Soil, Sequential extraction procedure, Zeien and Brümmer method

## Abstract

The aim of this paper is to assess the effect of liming and low doses of municipal sewage sludge (5%, 10%, 15% of the soil mass) on lead, chromium and nickel speciation in soil. The 420-day experiment was carried out in laboratory conditions. In all the samples lead, chromium and nickel concentration was determined with the ICP-AES method, while the content of those metals in different fractions was measured with the seven-step Zeien and Brümmer method, on the 30th and 420th days of the experiment. Sewage sludge doses significantly diversified lead, chromium and nickel amounts in the soil. The highest dose of sludge caused a significant increase, compared to the control, in the content of those metals. In the sludge the dominant forms of metals tested in the experiment were lead and chromium bound to organic matter (F4) as well as nickel bound to amorphous iron oxides (F5). Liming decreased the mobility of the metals in the soil.

Produced as a by-product during municipal wastewater treatment, sewage sludge is rich in organic matter and nutrients (Unsal and Ok 2001; Speir et al. 2003; Jouraiphy et al. 2005). The total amount of heavy metals in the sludge is not enough to assess their toxicity; another factor is their mobility, which is usually determined with the sequential extraction procedure (Glyzes et al. 2002; Amir et al. 2004; Wang et al. 2005; Jamali et al. 2007; Rao et al. 2008; Malinowska 2016). This method is based on extraction of mobile and stable forms, the latter being unavailable for plant uptake.

High quantities of heavy metals in soil can lower its fertility and, in consequence, yield quantity and quality (Fleißbach et al. 1994; Muchuweti et al. 2006). Some factors can influence mobility of heavy metals. According to Kabata-Pendias and Pendias (1999) plant roots can produce some substances which increase complexation and solubility of heavy metals. The most common of those substances affecting heavy metal forms are oxalic and acetic acids as well as different amino acids. Liming as well as quality and quantity of organic matter in the soil significantly affect speciation of heavy metals (Hsu and Lo 2001; Sánchez-Monedero et al. 2001). The experiment was carried out with various doses of sewage sludge applied to the soil. Because vegetation can affect heavy metal speciation in soil, no plants were grown in the pots. The study was conducted to determine whether organic matter in lime-stabilised soil would form complexes with heavy metals and whether a changing pH value would affect this process.

The aim of the paper is to assess the effects of liming and different doses of sludge on total concentration and speciation of lead, chromium and nickel in soil.

## Materials and Methods

The 420-day incubation experiment was conducted in laboratory conditions and pots were filled with 3 kg of soil, with a pH_KCl_ of 4.30. The soil was taken from the humus layer with the granulometric composition of silty light loamy sand (according to Polish Society of Soil Science classification 2008). Soil particle-size composition determined with the aerometric method was as follows: 1 to 0.1 mm—60%; 0.1 to 0.05 mm—7%; 0.05 to 0.02 mm—17%; 0.02 to 0.06 mm—10%; 0.06 to 0.002 mm—6%. The soil was collected from fields of local farmers. Before the experiment started, the concentration of some metals in the soil was as follows (mg kg^−1^): Pb—6.03; Cd—0.120; Cr—2.09; Cu—2.21; Zn—19.95; Ni—1.56. Carbon concentration in the soil was 10.1 g kg^−1^DM, and calcium concentration was 1.58 g kg^−1^DM, both of which being below the limits for loose soil treated with sewage sludge set by the Polish Ministry of the Environment Regulation on soil quality standards of 2002 (Journal of Laws 2002, item 1359). Once the soil was sieved through a mesh, the pots were filled and divided into two groups: one was not limed but the other was limed with CaCO_3_ of hydrolytic acidity Hh = 1. Next, the pots with limed and non-limed soil were left for 1 month with the moisture ranging from 50% to 60% of maximum water holding capacity. Applied at a dose of 3 g per pot, pure dehydrated CaCO_3_ of recognized analytical grade was provided by TechlandLab Limited, Tarnobrzeg, Polska.

Then, fresh municipal sewage sludge from the Mechanical–Biological Wastewater Treatment Plant in Siedlce was added in different doses of 150, 300 and 450 g, which constituted 5%, 10% and 15% of the dry mass of soil, respectively. Then, the contents were thoroughly mixed. In the sewage sludge total heavy metal concentration was as follows (mg kg^−1^): Pb—50.23; Cd—0.174; Cr—19.85; Cu—85.0; Zn—1120; Ni—50.14. It did not exceed the limits set by the Polish Minister of the Environment Regulation of 2010 (Journal of Laws 2010, item 924). Moreover, the sludge contained 25% of dry matter, total nitrogen content was 40.00 g kg^−1^, organic carbon concentration was 345 g kg^−1^, while pH_KCl_ was 6.8.

Two controls were used in the experiment: one without liming and without sludge, the other with liming (CaCO_3_) but without sludge. During the experiment moisture was kept at 50%–60% of maximum water holding capacity and the air temperature was 20–22°C. In the first part of the experiment soil samples were taken four times at 30-day intervals. This 120-day part of the experiment is referred to as ‘the first series’. Then no samples were collected for 180 days while the moisture and the temperature were as above. During the next period soil was sampled twice, with the first sample taken at the beginning and the other taken after 60 days. The second series lasted another 120 days, so the whole experiment was 420 days long. Soil samples were collected six times, after 30, 60, 90, 120, 360 and 420 days.

In all the samples total lead, chromium and nickel concentration was measured after dry mineralization at 450°C. Next 10 ml of hydrochloric acid (HCl) solution (1:1) was added to the crude ash and the contents of the porcelain crucible were evaporated in a sand bath to decompose carbonates, and to isolate silica. Using a hard filter, the contents, after adding 5 ml of 10% HCl, were filtered into a 100 ml conic flask which was then filled up to the mark with distilled water. Content of selected chemical elements in this solution was determined using Inductively Coupled Plasma*-*Atomic Emission Spectrometry (ICP-AES), while calibration was performed using standard Merck solutions.

An internal quality control procedure was used to verify the accuracy of the methods. Two measurements were taken for each series of samples with the recovery being within the 85%–115% range. The limit of detection for Pb, Cd and Ni was 0.01 mg kg^−1^.

In the soil taken on the 30th and 420th days and in the municipal sewage sludge the seven step Zeien and Brümmer method (1989) was used to measure the distribution of the metals in the fractions (Table 1).

**Table 1:** Sequential extraction of heavy metals with the Zeien and Brümmer method

Fraction	Name	Extraction reagent	Extraction time (h)	pH
F1	Easily soluble	1 mol NH_4_NO_3_ dm^−3^	24	Natural
F2	Exchangeable	1 mol CH_3_COONH_4_ dm^−3^	24	6.0
F3	Bound to MnO_x_	1 mol NH_2_OH·HCl dm^−3^ + 1 mol CH_3_COONH_4_ dm^−3^	0.5	6.0
F4	F_org_ bound to organic matter	0.025 mol C_10_H_22_N_4_O_8_ dm^−3^	1.5	4.6
F5	Bound to amorphous FeO_x_	0.2 mol (NH_4_)_2_C_2_O_4_ dm^−3^ + 0.2 mol H_2_C_2_O_4_ dm^−3^	4	3.25
F6	Bound to crystalline FeO_x_	0.2 mol (NH_4_)_2_C_2_O_4_ dm^−3^ + 0.2 mol H_2_C_2_O_4_ dm^−3^ + 0.1 mol C_6_H_8_O_6_ dm^−3^	0.5	3.25
F7	F_resid_ residual	Calculated as the difference between the total content of lead, chromium and nickel and the sum of the above determined fractions	–	–

Soil/solution ratio, 1 g:10 cm^3^

The results of Pb, Cr and Ni concentration were statistically analysed. The three-factor analysis of variance was used to evaluate differences between the effects of sewage sludge doses (A), liming (B) and the sampling days (C) (using the Statystica software, Version 10.0 StatSoft). Tukey’s HSD test was used to compare differences between means.

## Results and Discussion

**Fig. 1:**
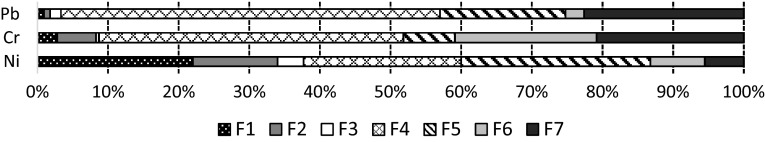
Percentage share of Pb, Cr and Ni fractions in their total content in sewage sludge: F1—easily soluble, F2—exchangeable, F3—bound to MnOx, F4—bound to organic matter, F5—bound to amorphous FeO_x_, F6—bound to crystalline FeO_x_, F7—residual

Sequential extraction of lead, chromium and nickel in the municipal sludge revealed a large variation in the distribution of those metals in the fractions (Fig. 1). Comparing all the fractions it was found that the highest amounts of lead and chromium (over 40% in both cases) were bound to organic matter (F4). Similarly, Álvarez et al. (2002) states that the organic fraction is dominant in sewage sludge. The highest percentage of nickel (26.81%) was bound to amorphous iron oxides (F5), with considerable amounts of this metal in the easily soluble (F1) and exchangeable (F2) fractions, both of them together constituting 34.03% of the total amount. There was much less chromium in those fractions (8.30% of the total amount) and an inconsiderable percentage of lead, standing at 1.78%.

The most abundant metal bound in the residual fraction (F7) was lead (22.63% of its total amount), with a lesser amount of chromium (20.86%), and a small amount of nickel (5.54%). Malinowska (2016) found that the residual fraction (F7) constituted as much as 35% of total copper content in municipal sewage sludge. In similar studies, using the Community Bureau of Reference method (BCR), Wang et al. (2006) found different content of metals in the residual fractions, depending on the origin of sewage sludge, while Álvarez et al. (2002) confirmed high mobility of nickel in sewage sludge.

Analysis of variance showed that sewage sludge (F = 7.2; p = 0.000), liming (F = 4.8; p = 0.031), and incubation time, as well as an interaction between those factors, significantly affected lead concentration in the soil (Table 2), ranging from 5.03 to 9.55 mg kg^−1^, while Kabata-Pendias and Pendias (1999) conclude that lead concentration is much higher for most soil types. On average, there was more lead in the non-limed soil than in limed soil. Additionally, liming did not affect lead concentration in the soil of the control pot. Overall, this concentration was significantly highest after 120 days and lowest after 60 days. Furthermore, Kabata-Pendias and Pendias (1999) point out that lead content in soil is dependent on mineralogical and granulometric composition of soil, properties of parent rock and on organic waste applied as fertiliser.

**Table 2:** Total content of lead, chromium and nickel (mg kg^−1^) in soil in the incubation experiment

Days	Without liming				Mean −Ca	With liming				Mean +Ca	Mean				Mean
	0	5%	10%	15%		0	5%	10%	15%		0	5%	10%	15%	
Pb															
30	6.13a	7.20ab	8.05b	7.50c	7.22	6.11a	7.55a	7.29ab	7.33a	7.07	6.12a	7.38a	7.67c	7.41b	7.15ab
60	5.84a	6.42bc	7.05c	7.51c	6.71*	5.92a	6.01c	6.53b	7.06a	6.38*	5.88a	6.21c	6.79c	7.28b	6.54c
90	5.33a	7.80a	7.93b	7.70c	7.19*	5.03b	7.06ab	7.75a	7.34a	6.80*	5.18c	7.43a	7.84bc	7.52b	6.99b
120	5.85a	7.73a	8.38ab	8.21bc	7.54*	5.09b	7.40a	8.04a	7.75a	7.07*	5.47bc	7.56a	8.21a	7.98a	7.31a
360	5.64a	6.17c	8.94a	9.55a	7.58*	5.75ab	6.25c	7.35a	7.02a	6.59*	5.69b	6.21c	8.14ab	8.28a	7.09ab
420	5.52a	6.74bc	8.59ab	8.92ab	7.45*	5.50ab	6.35bc	7.50a	7.42a	6.69*	5.51b	6.54b	8.04ab	8.17a	7.07ab
Mean	5.72	7.01*	8.16*	8.23*	7.28*	5.57	6.77*	7.41*	7.32*	6.77*	5.64C	6.89B	7.78A	7.78A	7.03
Cr															
30	2.60ab	3.47a	3.12c	3.78c	3.24	2.09d	3.69ab	3.17c	3.63c	3.15	2.35d	3.58b	3.15c	3.71d	3.20d
60	2.98a	3.62a	3.94ab	5.10a	3.19*	3.15ab	4.07a	4.41a	7.71a	4.84*	3.07a	3.85a	4.18a	6.41a	4.37a
90	2.77a	3.11a	4.40a	4.09bc	3.59	3.22a	4.10a	3.64bc	3.96c	3.73	3.00ab	3.61b	4.02ab	4.03c	3.66bc
120	2.55ab	3.14a	3.79b	4.19bc	3.42	2.84abc	3.52b	3.80ab	3.97c	3.53	2.70b	3.33c	3.80b	4.08c	3.48c
360	2.54ab	3.65a	3.75b	4.39b	3.58*	2.63bcd	3.45b	4.12ab	5.01b	3.80*	2.59bc	3.55bc	3.94b	4.69b	3.69b
420	2.21b	3.66a	3.87ab	4.50b	3.56*	2.56cd	3.78ab	4.05ab	4.99b	3.85*	2.39cd	3.72ab	3.96ab	4.75b	3.70b
Mean	2.61	3.44*	3.81	4.34*	3.55*	2.75	3.77*	3.87	4.88*	3.82*	2.68D	3.61C	3.84B	4.61A	3.68
Ni															
30	1.36a	1.91ab	1.86c	2.61ab	1.94	1.33abc	2.08ab	1.83c	2.11b	1.84	1.35bc	2.00a	1.85c	2.36bc	1.89c
60	1.41a	1.60a	1.68c	2.31b	1.75	1.36abc	1.69b	1.90bc	2.09b	1.76	1.39c	1.65b	1.79c	2.20c	1.76c
90	1.57a	1.86ab	2.69a	2.61ab	2.18	1.60ab	2.32a	2.09abc	2.41ab	2.11	1.59a	2.09a	2.39a	2.51b	2.15ab
120	1.52a	2.07a	2.17b	2.40ab	2.04	1.66a	1.94ab	2.41a	2.28b	2.07	1.59a	2.01a	2.29a	2.34bc	2.06ab
360	1.20a	1.85ab	2.01bc	2.75ab	1.95	1.11c	1.99ab	2.07abc	2.69a	1.97	1.16ab	1.92a	2.04b	2.72a	1.96b
420	1.25a	1.98ab	2.41ab	2.84a	2.12	1.18bc	2.01ab	2.31ab	2.80a	2.08	2.43a	2.00a	2.36a	2.82a	2.10a
Mean	1.39	1.88*	2.14	2.59*	2.00	1.37	2.01*	2.10	2.40*	1.97	1.38C	1.94C	2.12B	2.49A	1.99

Zero-control object, sewage sludge at the dose of 5%, 10%, 15% to dry mass of soil; Means for sewage sludge doses marked with upper case letters are significantly different (at p ≤ 0.05)

*Means in lines for liming (B), interaction between sewage sludge and liming (A × B) and interaction between incubation time and sewage sludge (C × B) marked with *asterisk* are significantly different (at p ≤ 0.05); Means in columns for incubation time (C), interaction between sewage sludge and incubation time (A × C) and sewage sludge, incubation time, and liming (A × C × B) marked with lower case letters are significantly different (at p ≤ 0.05)

All the experimental factors and their interactions caused significant differences between total chromium concentrations in the soil (Table 2). Analysis of variance showed that sewage sludge (F = 14.56; p = 0.000), liming (F = 6.8; p = 0.011) and incubation time (F = 8.2; p = 0.001) affected chromium concentration in the soil. Moreover, it was significantly highest after 60 days and lowest after 30 days. Concentration of this metal ranged from 2.09 to 7.71 mg kg^−1^ and increased with the sludge dose. It was still much lower than the limits concerning soil sludge utilisation set by the Polish Minister of Environment Regulation (Journal of Laws 2002, item 1359).

As a result of sewage sludge application nickel concentration in the soil significantly varied and ranged from 1.11 to 2.84 mg kg^−1^ (Table 2). The content of nickel in the present experiment did not exceed limits set by the Polish Minister of the Environment Regulation (Journal of Laws 2002, item 1359). Liming did not significantly affect nickel concentration in the soil. However, like in the case of other heavy metals discussed here, sewage sludge had a significant effect on this concentration. The highest dose of sewage sludge (15%) resulted in the highest increase in nickel concentration.

**Fig. 2:**
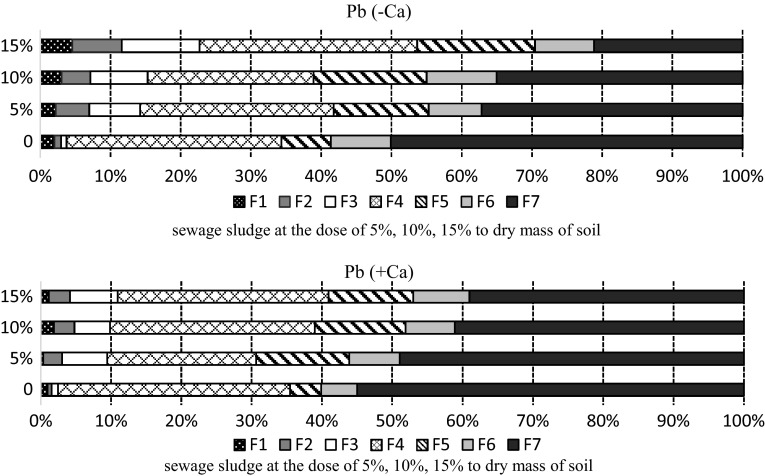
Percentage of lead fractions in the soil sampled after 30 days: F1—easily soluble, F2—exchangeable, F3—bound to MnO_x_, F4—bound to organic matter, F5—bound to amorphous FeO_x_, F6—bound to crystalline FeO_x_, F7—residual

At the beginning of the experiment, 30 days after sewage sludge had been applied, the seven-step sequential extraction procedure was carried out. It was found that lead content in the mobile fractions was low, but it increased along with the dose and was higher in the non-limed soil than in the limed soil (Fig. 2). In the soil where the dose was highest (15%), the amount of lead in the mobile fractions constituted 11.61% of the total content of this metal.

Contrary to the mobile fractions, in the residual fraction (F7) lead content decreased when the dose of sewage sludge was increased, although the biggest amount of this metal was found in this fraction. Like in the case of the mobile fractions, content of lead bound to iron oxides increased when doses of sludge was increased. The amount of lead in this fraction was higher in non-limed soil than in limed soil, again similarly to the mobile fractions.

**Fig. 3:**
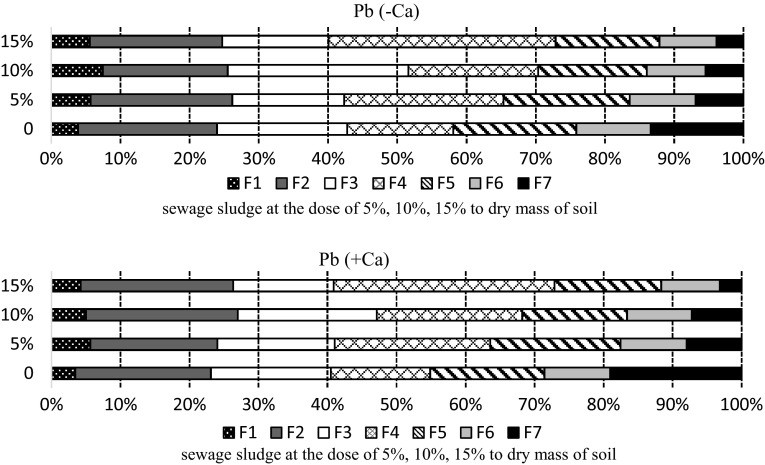
Percentage of lead fractions in the soil sampled after 420 days: F1—easily soluble, F2—exchangeable, F3—bound to MnOx, F4—bound to organic matter, F5—bound to amorphous FeO_x_, F6—bound to crystalline FeO_x_, F7—residual

Lead bound to the organic fraction constituted 28% of the total amount of lead, both in the limed and non-limed soil. According to many publications lead is one of the least mobile metals, being easily absorbed and forming sparingly soluble organic and inorganic compounds (Kabata-Pendias and Pendias 1999). However, 420 days after the beginning the experiment lead speciation results showed that this metal had been transformed into easily soluble forms with higher mobility and higher toxicity (Fig. 3), a similar change being confirmed by Domańska (2006). At that time, the combined amount of lead in the soluble (F1) and exchangeable (F2) fractions increased by several times, standing at over 20%. Similarly, the percentage of lead bound to manganese oxides (F3) and to iron oxides (F5 and F6) increased too.

Likewise, Kabata-Pendias and Pendias (1999) and Wang et al. (2006) say that the highest amounts of lead are mainly bound to iron oxides and organic matter. On the whole sewage sludge application decreased the amount of this metal in the organic fraction but the highest amount of lead was noted in the soil with the highest dose of sludge applied. In the same manner, sludge application strongly decreased the amount of this metal in the residual fraction (F7), with lower amounts in the soil where higher doses were applied. In the soil with 15% of the sludge applied, the percentage of this form of lead after 420 days was only 4.5% of the total amount. Studying copper concertation in soil enriched with different amounts of sewage sludge, Malinowska (2016) found similar results. She noted that the share of copper in the residual fraction (F7) had decreased fourfold, while the share of this metal bound to amorphous FeO_x_ fraction (F5) increased.

**Fig. 4:**
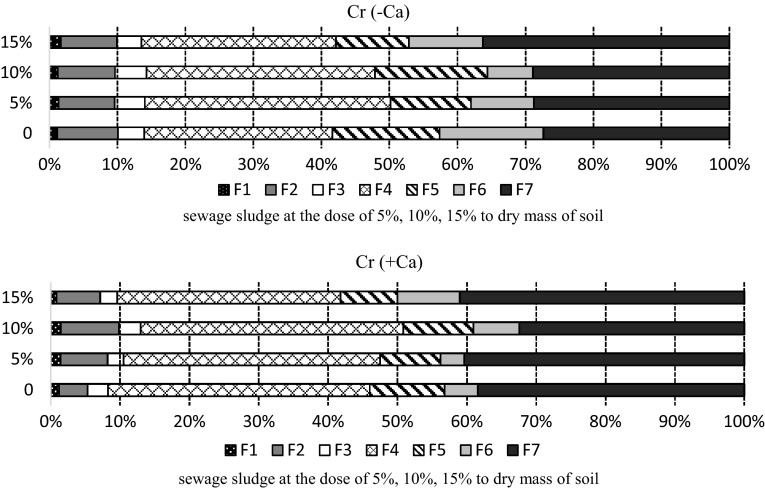
Percentage of chromium fractions in the soil sampled after 30 days: F1—easily soluble, F2—exchangeable, F3—bound to MnOx, F4—bound to organic matter, F5—bound to amorphous FeO_x_, F6—bound to crystalline FeO_x_, F7—residual

At the beginning of the experiment the sequential extraction procedure showed that dominant forms of chromium were those bound to the organic (F4) and residual (F7) fractions (Fig. 4). Percentage of this metal was much higher in the limed soil, with F4—36.2% and F7—38.1%, than in the non-limed soil, with F4—31.5% and F7—30.4%. In the non-limed soil there was more chromium bound to iron oxides (F5 and F6) and manganese oxides (F3) than in limed soil.

Calcium carbonate application lowered the content of chromium in the mobile fractions, i.e. F1 and F2, and thus the aggregated content of both fractions stood, on average, at 9.12% in the limed soil, and at 12.61% in the non-limed soil.

**Fig. 5:**
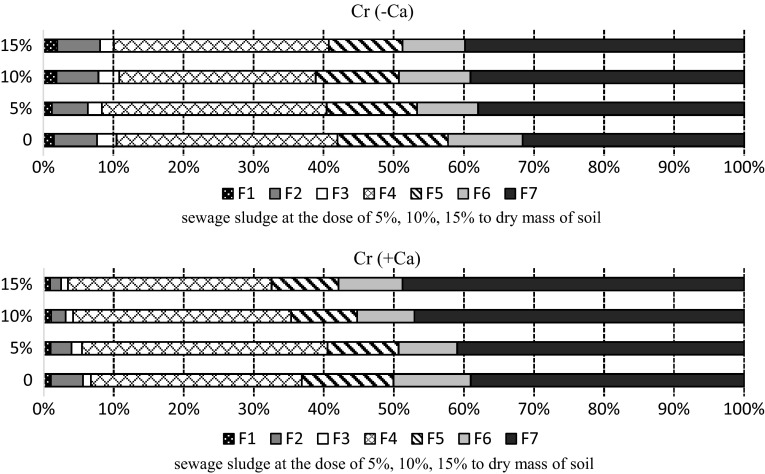
Percentage of chromium fractions in the soil sampled after 420 days: F1—easily soluble, F2—exchangeable, F3—bound to MnOx, F4—bound to organic matter, F5—bound to amorphous FeO_x_, F6—bound to crystalline FeO_x_, F7—residual

In the soil after 420 days of the experiment the content of strongly bound chromium in F5, F6 and F7 fractions increased, in particular in the limed soil. The aggregate content of the metal in those fractions amounted to 40% (Fig. 5). In the latter part of the experiment, compared to the first series, there was less chromium bound to manganese oxides (F3) and in the mobile fractions. Chromium in those fractions constituted, on average, 3.78% of the total amount in the limed soil, with 7.51% in the non-limed soil but Kalembasa and Pakuła (2009), using the BCR sequential extraction procedure to analyse soil in East-Central Poland, found that 1.59%–3.58% of the total chromium content was in the easily soluble fraction. Finally, it should be noted that there was a high amount of chromium bound to organic matter (F4), on average more than 30%.

**Fig. 6:**
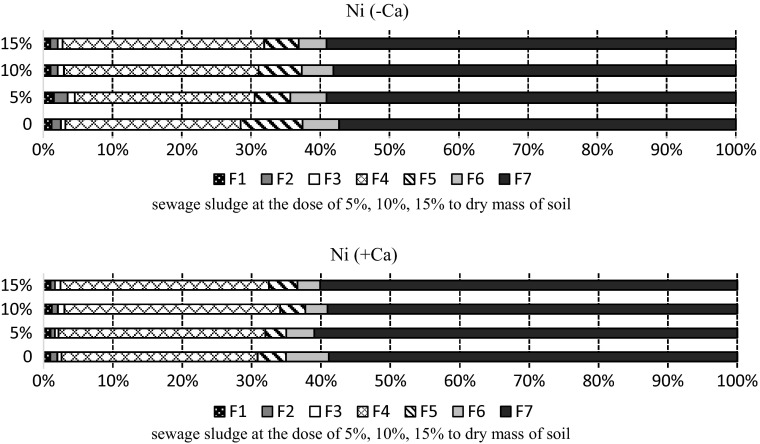
Percentage of nickel fractions in the soil sampled after 30 days: F1—easily soluble, F2—exchangeable, F3—bound to MnOx, F4—bound to organic matter, F5—bound to amorphous FeO_x_, F6—bound to crystalline FeO_x_, F7—residual

The sequential extraction procedure used to analyse the soil treated with sewage sludge showed that the content of nickel in the seven fractions varied and was correlated with sludge doses and the time when samples were taken (Fig. 6). The highest amount of this metal was in the residual fraction (F7), measuring, on average, 58.4% in the non-limed soil, and at 59.8% in the limed soil. A considerable amount of nickel, increasing with the increase of the dose, was in the organic fraction (F4). In the non-limed soil treated with the highest dose of sludge this metal in the organic fraction constituted 29.12% of the total nickel content, while in the control it was 25.31%. In the limed soil these values were 28.31% and 30.06%, respectively. Nickel content in the mobile fractions was small, being higher in the non-limed soil (2.56%) than in the limed soil (1.84%).

**Fig. 7:**
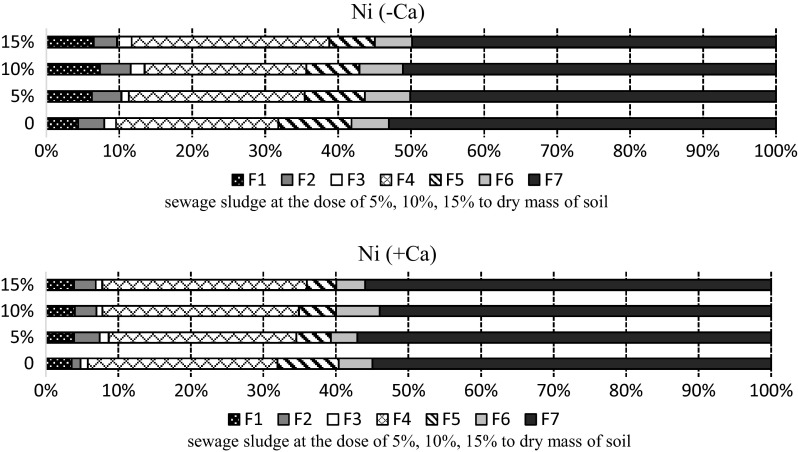
Percentage of nickel fractions in the soil sampled after 420 days: F1—easily soluble, F2—exchangeable, F3—bound to MnOx, F4—bound to organic matter, F5—bound to amorphous FeO_x_, F6—bound to crystalline FeO_x_, F7—residual

After 420 days of the experiment the amount of nickel available to plants increased threefold compared to the amount in the sample taken on the 30th day (Fig. 7). The highest content of available nickel in the mobile fractions (11.57%) was in the non-limed soil with the mean dose of sewage sludge (10%) applied. After 420 days the content of nickel in other fractions decreased, with the exception of the fraction where nickel is bound to manganese oxides (F3).

Dominant amounts of nickel were in the residual fraction, with 55.6% in the limed soil and 51.06% in the non-limed soil. Correspondingly, Andersen et al. (2002) showed that in mineral soil over 60% of nickel is in the residual fraction, which means that most of this metal is immobile and tightly bound.

In sewage sludge treatments, the percentage of lead, chromium, and nickel fractions within the total amounts of those metals can be put in the following order, from highest to lowest:

Pb: F4(53.6) > F7(22.6) > F5(17.8) > F6(2.65) > F3(1.56) > F1(0.926) > F2(0.854);

Cr: F4(43.1) > F7(20.9) > F6(20.1) > F5(7.30) > F2(5.49) > F1(2.81) > F3(0.418);

Ni: F5(26.8) > F4(22.3) > F1(22.0) > F2(12.0) > F6(7.70) > F7(5.54) > F3(3.67).

In the incubation experiment various doses of sewage sludge significantly affected the amount of the heavy metals in the soil. On the whole, lead, chromium and nickel content increased with the increase of sewage doses. Distribution of the heavy metals in the soil fractions was dependent on sewage doses and on liming.

Out of all the metals, lead, followed by nickel, was fastest when it comes to redistribution to more mobile fractions, potentially becoming more toxic to plants. In the soil sampled after 420 days of the experiment the amount of lead in the residual fraction decreased by several times. It also decreased when the dose of sludge increased. At the same time an increase of stable forms of chromium, in the fraction bound to FeO_x_, (F5 and F6) and in the residual fraction (F7), was noted. Throughout the experiment there were no major changes in the forms of nickel.
